# Designing modulators of monoamine transporters using virtual screening techniques

**DOI:** 10.3389/fphar.2015.00223

**Published:** 2015-09-29

**Authors:** Ole V. Mortensen, Sandhya Kortagere

**Affiliations:** ^1^Department of Pharmacology and Physiology, Drexel University College of Medicine, Philadelphia, PA, USA; ^2^Department of Microbiology and Immunology, Drexel University College of Medicine, Philadelphia, PA, USA

**Keywords:** dopamine transporter, modeling and simulations, monoamine transporters, norepinephrine transporter, serotonin transporter, hybrid structure based screening, virtual screening

## Abstract

The plasma-membrane monoamine transporters (MATs), including the serotonin (SERT), norepinephrine (NET) and dopamine (DAT) transporters, serve a pivotal role in limiting monoamine-mediated neurotransmission through the reuptake of their respective monoamine neurotransmitters. The transporters are the main target of clinically used psychostimulants and antidepressants. Despite the availability of several potent and selective MAT substrates and inhibitors the continuing need for therapeutic drugs to treat brain disorders involving aberrant monoamine signaling provides a compelling reason to identify novel ways of targeting and modulating the MATs. Designing novel modulators of MAT function have been limited by the lack of three dimensional structure information of the individual MATs. However, crystal structures of LeuT, a bacterial homolog of MATs, in a substrate-bound occluded, substrate-free outward-open, and an apo inward-open state and also with competitive and non-competitive inhibitors have been determined. In addition, several structures of the *Drosophila* DAT have also been resolved. Together with computational modeling and experimental data gathered over the past decade, these structures have dramatically advanced our understanding of several aspects of SERT, NET, and DAT transporter function, including some of the molecular determinants of ligand interaction at orthosteric substrate and inhibitor binding pockets. In addition progress has been made in the understanding of how allosteric modulation of MAT function can be achieved. Here we will review all the efforts up to date that has been made through computational approaches employing structural models of MATs to design small molecule modulators to the orthosteric and allosteric sites using virtual screening techniques.

## Introduction

The family of sodium coupled plasma membrane monoamine transporters (MATs) include the serotonin (SERT), dopamine (DAT), and norepinephrine (NET) transporters (Torres et al., 2003; Kristensen et al., 2011). They are critically involved in regulating monoaminergic neurotransmission through the reuptake of their respective neurotransmitter. They are all expressed in the presynaptic neuron from which their respective monoamine neurotransmitter (MA) is synthesized and released. As a consequence MAT mediated reuptake of MAs is also believed to be important for MA recycling and repackaging in the presynaptic monoaminergic neuron (Torres et al., 2003). All MATs are pharmacological targets of some of the most psychoactive compounds in clinical use. These include inhibitors such as tricyclic antidepressants (TCAs), selective serotonin reuptake inhibitors (SSRIs), and psychostimulants such as amphetamine and cocaine.

## Early Structure/Function Studies

The MATs are members of the neurotransmitter:sodium symporter (NSS) SLC6 family of transporters that also include GABA transporters (Kristensen et al., 2011). Immediately upon the identification of the genes of the MATs and related transporters within the NSS family it was realized that they were 12 transmembrane domain (TM) containing proteins. Early structure function studies confirmed through biochemical assays that the N- and C-termini of the proteins were intracellular (Kristensen et al., 2011). Functional characterization also established that they are sodium-dependent symporters utilizing the sodium ion gradient established by sodium/potassium ATPases to transport their respective substrates against their gradients. The transporters are also dependent on extra-cellular chloride, though the understanding of the role of chloride in the transport process is limited (Kanner, 1989; Rudnick, 1998). The transport process is believed to proceed through a classical alternating access mechanism with the transporter going through several different conformations that include states in which the substrates are occluded from the intracellular and extracellular environment (Krishnamurthy et al., 2009; Penmatsa and Gouaux, 2014). Expression of the transporters in *Xenopus* oocytes established that substrate translocation is electrogenic and involves the movement of sodium and chloride ions (Sonders and Amara, 1996). Some of the ion fluxes are coupled to the transport cycle but these currents are larger than predicted from stoichiometric calculations. In addition uncoupled currents have also been demonstrated that display similarities to an ion-channel like flux. The early structure function studies pointed to TM1 as being critical for substrate interactions—in particular an aspartate centrally located within TM1 (Kitayama et al., 1992; Barker et al., 1999). Studies on inhibitor interaction also found evidence for an important role for residues in TM1 and 3 (Barker et al., 1998; Larsen et al., 2004). Though these studies provided highly significant progress in the molecular understanding of transporter function and ligand interactions they were limited by the lack of high resolution three dimensional (3D) structures to guide additional mechanistic studies.

## Crystal Structures of LeuT

A dramatic change in our structural understanding of the MATs occurred with the elucidation of the 3D structure of LeuT, a bacterial leucine transporter homolog of the MATs. The first structure that was elucidated was of LeuT bound to its substrate leucine occluded from the extracellular and intracellular environment (Yamashita et al., 2005). The structure also revealed the location of two sodium ion binding sites. In this crystal structure, the transporter was a homo-dimer and each monomer consisted of 12 TMs with some TMs being discontinuous. The protein consisted of an intriguing pseudo twofold axis of symmetry formed by TM1–5 and TM6–10 respectively that can be superimposed on each other. This intriguing novel fold, that was unique at the time of elucidation has now been found in several other unrelated transporter families and is therefore not restricted to only the NSS family (Penmatsa and Gouaux, 2014). The crystal structures also highlighted that domains TM1 and 6 and TM3 and 8 forms and defines the inner core translocation pathway. This is in agreement with the structure/function studies that were performed prior to the structure determinations that demonstrated a role for TM1 and 3 in both substrate and inhibitor interactions. The substrate leucine was occluded from the extracellular and intracellular space by a gate structure formed by both ionic and hydrophobic interactions between specific residues. Following the initial publication of the LeuT transporter in the out-ward facing substrate-occluded conformation several other conformational states of this transporter have now been elucidated. These structures include structures with a non-competitive TCA bound to an extracellular vestibule above the proposed extracellular gate (Singh et al., 2007; Zhou et al., 2007) and a structure of a competitive inhibitor tryptophan bound to a forced open conformation of LeuT (Singh et al., 2008). Finally, structures have been elucidated of LeuT in substrate-free open and inward-facing conformations (Krishnamurthy and Gouaux, 2012).

## Crystal Structure of *Drosophila* DAT

A further major step forward in our structural understanding of MAT function was achieved by the elucidation of an actual MAT as the *Drosophila* DAT was crystalized and a high resolution structure was obtained (Penmatsa et al., 2013). This structure also provided support for the use of LeuT structures as models for MAT studies as it was found that the DAT structure was similar to LeuT. Some minor but interesting differences were found in the C-terminal part of the protein as a kink was observed in TM12 and the intracellular C-terminus was found to form a helix that interacted with the first intracellular loop. In support of previous biochemical studies (Hong and Amara, 2010) the DAT structure also revealed specific interactions with membrane lipids including cholesterol. Finally, additional *Drosophila* structures have recently been determined revealing substrate, psychostimulant (Wang et al., 2015), and antidepressant binding sites (Penmatsa et al., 2015).

## Functional Studies Based on MAT Models

With the availability of the bacterial LeuT and *Drosophila* DAT structures, several seminal modeling and structure functions studies based on the crystal structures have now enabled a much clearer understanding of several aspects of mammalian MAT function. A review by Manepalli et al. (2012), highlighted the various conformational states of LeuT and the utility of these crystal structures to understand the structure-function and dynamics of SERT, DAT and NET. The models proposed based on the LeuT crystal structures were used to develop hypothesis to understand the role of these MATs in normal and disease states (Vaughan and Foster, 2013). These models of MATs were instrumental in identifying the various functional sites including the substrate, inhibitor, ion, allosteric binding sites and to design selective modulators as described below.

### Substrate Binding Site

For all three MATs, modeling studies and structure/functions studies suggest that there is significant overlap between the leucine binding site in LeuT referred to as S1 and the monoamine binding sites in the MATs. In fact, several of the residues that form the binding site are conserved. Extensive structure function studies have been carried out on SERT and DAT that confirmed the importance of an aspartate in TM1 (Beuming et al., 2008; Celik et al., 2008; Indarte et al., 2008; Wang et al., 2012; Koldso et al., 2013). In addition, these studies also revealed the involvement of residues in TM3 and 8 for the interaction with serotonin (Celik et al., 2008). For DAT, the corresponding residues that were engaged in the interaction with dopamine were identified through modeling and experimental studies (Beuming et al., 2008). Furthermore, though difficult to verify experimentally, these studies also demonstrated an interaction between the substrates and the backbone carbonyls of the unwound region of TM1 and 6.

### Ion Binding Site

It is well established that all MATs are sodium dependent utilizing the gradient set up by the sodium/potassium ATPases to drive the transport of their individual substrates (Kanner, 1989; Rudnick, 1998). The mechanism of the coupling of sodium flux and substrate uptake is not well established. It was therefore highly significant that the LeuT structures revealed some information regarding the interaction of LeuT with sodium as two sodium ions were found to be co-crystalized. One of the sodium ions was found to interact directly with the substrate leucine and supports the hypothesis of direct coupling between sodium and substrate binding. Because the residues found to coordinate this central sodium ion in LeuT are conserved among the SLC6 transporters, it also suggests a common mechanism for all transporters in their engagement of the sodium gradients to drive substrate transport. Providing experimental support to explore mechanistic questions regarding the role of the sodium ions in facilitating transport are made difficult given their central and conserved role. A recent biophysical modeling study used extensive simulations to explore the conformational changes that occur following sodium binding. This study revealed a role for sodium in facilitating the closing of the extracellular gate formed by a central phenylalanine and the tilting of central TMs to enable the inward movement of the substrate (Zomot et al., 2015).

Different from the sodium dependent LeuT, all MATs are sodium and chloride dependent. Therefore there was no obvious information in the LeuT structures regarding the role of chloride in MAT transporter function. However, two independent studies did take advantage of the LeuT structure to identify a potential site of chloride interaction in the sodium and chloride dependent MATs. Comparing the sequence alignments of chloride dependent and independent NSS transporters, a negatively charged glutamate residue in TM7 was identified in the chloride independent transporters and it was suggested this negative charge provided by a glutamate residue has the same function as the negative charge provided by the chloride ion (Forrest et al., 2007; Zomot et al., 2007). Furthermore, the LeuT structure put this charged residue close to one of the sodium ion binding sites suggesting that the negative charge is directly linked to and cooperates with the sodium in all NSS transporters to facilitate transport. The role of this residue was further investigated experimentally by replacing the corresponding residue in chloride dependent transporters with glutamate resulting in chloride independent transporters and *vice versa* (Zomot et al., 2007).

### Gates

The first LeuT structure was in a conformation of the transporter with the substrate occluded from both the extra- and intra-cellular space (Yamashita et al., 2005). It therefore immediately suggested the location of protein domains involved in shielding and occluding the substrate during translocation. These domains are different from classical gates associated with ion-channels but the term “gate” will be used to discuss these domains in this review. In the out-ward facing conformation, an extracellular vestibule is positioned above the occluded substrate that provides access to the extracellular environment. At the bottom of this vestibule a domain forms the gate that occludes the substrate. This domain is formed by a conserved pair of charged residues forming a putative salt bridge (Pedersen et al., 2014) and further down, immediately below an additional seal is formed by a structure of two aromatic residues. The access to the occluded substrate binding site from the intracellular environment is hindered by a more complex thicker domain formed by TMs 1, 6, and 8. Another pair of charged residues is located below this domain forming an additional salt bridge that in MATs have been experimentally examined and shown to affect the conformational states of the transporters (Kniazeff et al., 2008).

### Inhibitor Binding Site

Most known inhibitors of MATs display competitive inhibition characteristics and a significant overlap between the substrate and inhibitor binding sites is therefore expected. Several extensive modeling studies have examined the site of interaction between antidepressants including TCAs and SSRIs with SERT and NET (Andersen et al., 2009, 2010, 2011, 2014; Koldso et al., 2010; Sinning et al., 2010; Severinsen et al., 2012, 2014; Wang et al., 2012) and psychostimulants including cocaine and its analogs with DAT (Beuming et al., 2008; Indarte et al., 2008; Loland et al., 2008; Bisgaard et al., 2011). Not surprisingly, these studies point to similar structures at the S1 site that are involved in substrate binding to also be important for interactions with all these above ligands.

Identification and characterization of the above determined functional sites serves as an important step toward designing small molecule modulators to treat disorders associated with the dysfunction of MATs. In the past, most drugs were discovered either through historical knowledge of natural products, serendipity, repurposing or through screening of small libraries of compounds using *in vitro* high throughput screening techniques. Although these methods yield useful lead compounds, the process is self-limiting, expensive and tedious. Virtual screening techniques have bridged the gap in terms of time and efforts to improve the early discovery process and to yield quality lead molecules.

## Virtual Screening and Ligand Design

Virtual Screening techniques have gained recognition as a useful and economical tool to screen millions of compounds in early drug discovery. Virtual screening in combination with *in vitro* and *in vivo* validation has the potential to improve the chances of finding better lead compounds. Some well-known examples of drugs that were designed using virtual screening techniques include the HIV integrase inhibitor raltegravir which was approved by FDA in 2007 to treat AIDS (Perryman et al., 2010). Tirofiban—a GP IIb/IIIa antagonist with anticoagulant properties has been used to treat myocardial infarction and was designed using an *in silico* virtual screening scheme and further optimized for drug like properties (Hartman et al., 1992). Most recently, we have developed novel antimalarial pyrazole amide class of compounds that disrupt the ion homeostasis in the malaria parasite leading to its death (Vaidya et al., 2014). This series of compounds were identified using our platform screening technology called the hybrid structure based (HSB) method (Kortagere et al., 2010). The fast killing rate induced by low picomolar concentrations of the pyrazole amide molecule along with its drug-like properties are key differentiators that has led to its inclusion in the discovery pipeline by Medicines for Malaria Venture (Vaidya et al., 2014). Other successful lead compounds that were designed using the HSB method include an atypical dopamine D3 receptor agonist that has shown significant promise in treating L-dopa induced dyskinesias and motor impairment in Parkinson’s disease in rodent models (Kuzhikandathil and Kortagere, 2012; Simms et al., 2015). These and other successful stories reported in literature (Geyer et al., 2005; Agarwal and Fishwick, 2010) have provided credibility to the use of virtual screening techniques in drug discovery.

Structural information for most members of the human SLC6 family is unavailable, however, computational modeling and virtual screening has contributed immensely to designing new inhibitors. These include SSRIs (Nolan et al., 2011), selective NET inhibitors (Schlessinger et al., 2011), triple uptake inhibitors (Kim et al., 2009), and most recently allosteric modulators of transporter function (Kortagere et al., 2013). Virtual screening has been broadly classified into ligand-based and structure-based methods (Kortagere et al., 2012), but the use of hybrid methods such as the hybrid structure-based method of screening have also gained importance (Kortagere and Welsh, 2006). Ligand based methods have used well known ligands to build quantitative structure activity relationship (QSAR) models to design congeneric series of MAT inhibitors (Schlessinger et al., 2011). Most significant among them were the 3D-QSAR and comparative molecular field analysis (CoMFA) methods that were used to derive several high affinity ligands primarily for DAT (Kulkarni et al., 2004; Christensen et al., 2007) and some triple uptake inhibitors that had nearly equal affinity for all three MATs. Some studies also employed ligand based pharmacophore methods to derive high affinity molecules by using the pharmacophores generated out of GBR-12909 and tropane analogs and screening the NCI database (MacDougall and Griffith, 2008). These early QSAR based screening models were well suited to design ligands due to lack of crystal structures of the human NSS/SLC6 protein family. However, crystal structures of LeuT from the bacterium *Aquifex aeolicus* in various states including outward open form, occluded form, in complex with various other amino acids (Singh et al., 2008), sugars (Quick et al., 2009), and drug molecules (Zhou et al., 2007, 2009) have provided suitable templates for modeling the corresponding conformational states of the human MATs. Structure based methods including receptor pharmacophore models and molecular docking methods have taken advantage of these LeuT based models for virtual screening of MAT inhibitors.

## Screening Inhibitors to Orthosteric S1 Binding Site

**FIGURE 1:**
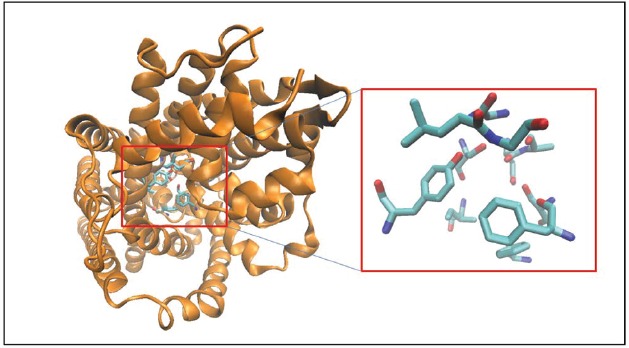
**Crystal structure of LeuT is shown in cartoon format and colored orange.** The binding site residues forming the S1 pocket (Singh et al., 2008; see text for details) are shown as licorice sticks and colored atom t ype (C-cyan, N-blue, O-red).

All MATs are considered to have a well-conserved primary binding pocket commonly referred to as the S1 pocket. The S1 pocket is the Leucine binding site derived from the LeuT crystal structures and was also validated using biochemical mutation studies in other MATs as described above for the substrate and inhibitor binding site. The S1 pocket (Figure 1) is lined by residues N21, A22, G24, L25, G26, Y108, I359, F253, S256, F259, S355 in the LeuT crystal structure bound to Leucine (pdb code: 3F3E; Singh et al., 2008). Virtual screening techniques using receptor pharmacophore models of the SERT S1 pocket has led to the design of novel SSRIs and antidepressant molecules (Nolan et al., 2011, 2014). A study by Schlessinger et al. (2011) utilized molecular docking and virtual screening techniques to screen the KEGG DRUGs (Kanehisa et al., 2010) database to design novel NET ligands binding to the S1 site of the transporter.

## Allosteric Binding Sites

For several decades a puzzling activity has been noted for some ligand interactions with SERT. It was demonstrated that when performing dissociation studies some ligands would have the rather unexpected effect of slowing the dissociation of other ligands (Chen et al., 2005; Neubauer et al., 2006; Zhong et al., 2012). It was speculated that this was caused by these ligands interacting with a second site that acted through an allosteric mechanism to affect the primary central S1 ligand binding site described above that overlaps with the substrate binding site. Structures with TCAs bound to LeuT exist and have provided a mechanism for these observations (Singh et al., 2007; Zhou et al., 2007). It is important to note that TCAs inhibit LeuT non-competitively and significant differences must exist in the low affinity interaction between TCAs and LeuT compared with the high affinity competitive interaction of TCAs with SERT. As a consequence, even though these structures reveal a high resolution picture of a TCA binding site in LeuT it is not likely that this site is similar to the high affinity TCA binding site in SERT. Indeed, in the LeuT structure co-crystalized with TCA, the TCA was found to be located above the extracellular gate-like structures that were described above to occlude the substrate binding site from the extracellular environment and therefore, also different from the substrate/inhibitor binding site (S1) described above that has been validated experimentally. In further structural support of this notion that the high affinity inhibitor binding site in MATs overlap with the central substrate binding site, it was found in all the most recent structures of the antidepressant sensitive *Drosophila* DAT that all examined inhibitors were bound to central structures overlapping with the dopamine binding site (Penmatsa et al., 2013, 2015; Wang et al., 2015). On the other hand, the observation of TCA binding to LeuT at a secondary site was tempting to be taken as evidence for an allosteric site and a recent study has explored how this secondary TCA site could relate to the allosteric interaction of some SERT ligands (Plenge et al., 2012). The study mutated residues in similar structures in the extracellular vestibule of mammalian SERT that was found to interact with TCA in LeuT. This reduced the above described allosteric effects on ligand dissociation from the primary substrate and inhibitor binding site located more centrally in SERT and supports the hypothesis that SERT ligands can modulate function through interactions with secondary sites.

**FIGURE 2:**
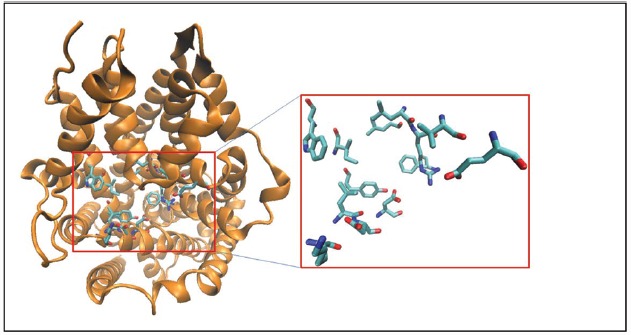
**Crystal structure of LeuT is represented in cartoon format and colored orange.** The binding site residues forming the TCA/S2 pocket (Nolan et al., 2011; see text for details) are shown as licorice sticks and colored atom type (C-cyan, N-blue, O-red). The TCA pocket is adjacent to the S1 pocket in the crystal structure.

The TCA binding pocket in LeuT [also referred to as the S2 site in some studies (Quick et al., 2009)] has been further characterized functionally as an allosteric site to the orthosteric binding site. The TCA binding pocket is proximal to the S1 site and is networked through a series of hydrogen bonds, therefore, conformational changes induced by TCAs at this site affect the translocation pathway. The TCA binding pocket (Figure 2) in the LeuT structure is formed by residues from TM1 (L25, L29, R30, V33, E37), TM3 (Y107, I111, W114), TM6 (F253), and TM10 (K398, L400, D401, D404) domains and is well conserved across all MATs (Nolan et al., 2011). Finally, a novel class of MAT modulators has been isolated from a marine *Conus marmoreus* snail. The Chi-conotoxin was found to specifically inhibit NET function in a non-competitively manner and structure/function studies suggested an interaction between this peptide and the extracellular vestibule (Bryan-Lluka et al., 2003; Paczkowski et al., 2007). Taken together, these studies opens up the possibility of targeting MATs in novel ways as ligands that specifically interact with structures outside the substrate binding site can have allosteric modulatory effects on MAT/ligand interactions and MAT function.

A few studies have directly targeted the TCA/S2 pocket for screening novel ligands. The study by Indarte et al. (2008) screened a library of 140,000 molecules against the TCA/S2 binding pocket of DAT to derive novel chemotypes that could competitively inhibit the binding of cocaine to the S1 site. In a similar study on the TCA/S2 binding pocket of SERT, virtual screening of the ZINC database led to the design of two novel SERT ligands (Manepalli et al., 2011). Improvements in computational resources and the availability of large combinatorial chemistry libraries from various vendors has also led to improvements in the screening technologies. A study by Gabrielsen et al. (2014) used a combined library of 3.24 million molecules to design novel SERT ligands using a variety of *in silico* techniques including 2D and 3D similarity, 3D pharmacophore and flexible docking methods. All these success stories suggest that structure based virtual screening is a powerful tool to design small molecule inhibitors to MATs, despite having no direct structural information on any of the MATs (except for the very recent *Drosophila* DAT) and a template identity less than 30%.

## Other Allosteric Pockets

**FIGURE 3:**
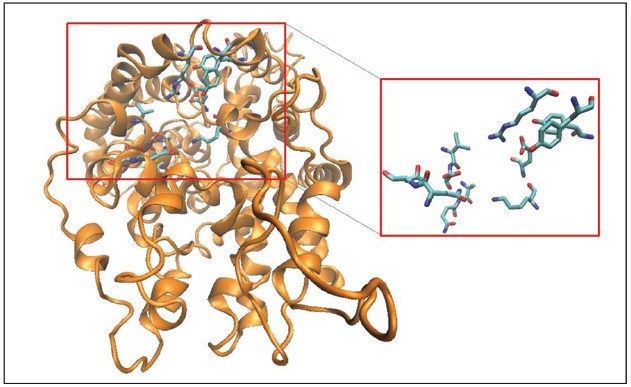
**Three-dimensional structural model of hSERT is represented in cartoon format and colored orange.** The binding site residues forming the A1 allosteric pocket (Kortagere et al., 2013; see text for details) are shown as licorice sticks and colored atom type (C-cyan, N-blue, O-red). The allosteric pocket is distinct from both the S1 and the TCA/S2 pockets and is positioned close to the extracellular region.

In addition to the well-known S1 and the allosteric TCA/S2 binding pockets of MATs, we have utilized molecular dynamics simulations and comparative genomics techniques to identify allosteric pockets outside the translocation pathway of MATs that when engaged can specifically modulate the binding of known transporter ligands (Kortagere et al., 2013). The hybrid structure based screening platform (Kortagere and Welsh, 2006) was used to design these allosteric modulators. Beginning with the 3D model of SERT structure immersed in a POPC membrane model, the HSB method incorporates dynamic conformational changes to screen for allosteric pockets. One such pocket that is conserved across all MATs, is a pocket (Figure 3) formed by TM1, TM6, TM10, TM11, and EL6 domains that we refer to as A1 and that is lined by residues Q111, N112, I327, D328, A331, Q332, K490, E494, R564, Y568, and Y572 in SERT. A four point receptor pharmacophore was developed using the residues D328, K490, E494 and Y568. The pharmacophore was used to screen a custom library of 3 million compounds leading to the design of ATM7—an allosteric modulator of SERT that binds to this novel site and elicits a conformational change that results in the facilitation of serotonin uptake and potentiates MDMA-elicited reversed transport. Among other compounds discovered through this screen are compounds that interact at novel A1 allosteric site and modulate the interaction of MATs with classical inhibitors including antidepressants and psychostimulants.

## Conclusion

Monoamine transporters play an important role in the brain by actively translocating MAs thereby modulating numerous physiological processes that are regulated by MAs. In addition, these plasma membrane transporters bind to a variety of compounds that achieve their effect through the inhibition of the monoamine uptake. These compounds include psychostimulant drugs and antidepressants that are therapeutically relevant and illicit drugs such as cocaine and methamphetamine. Dysfunctions of MATs are implicated in a variety of disorders including depression, mania, addiction and cognition suggesting their key role as therapeutic targets. Despite advances in protein crystallography techniques, 3D structure of only a few members of the NSS family that include MATs are available. Computational techniques including homology modeling, molecular dynamics simulations and virtual screening techniques have resulted in a better understanding of the structure-activity relationships of various inhibitors and substrates with MATs and the design of novel chemotypes to the orthosteric and allosteric pockets. Improvements in screening technologies and the availability of larger chemical libraries have also significantly improved the quality of these ligands resulting in highly efficacious and sub-type selective ligands. Since MATs can be significant targets for both disease modifying and symptomatic treatments of many psychiatric diseases, novel chemotypes that can uniquely target these transporters with improved side effect profiles will be highly desirable.

### Conflict of Interest Statement

The authors declare that the research was conducted in the absence of any commercial or financial relationships that could be construed as a potential conflict of interest.
